# Launching the "*Journal of Biomedical Discovery and Collaboration*"

**DOI:** 10.1186/1747-5333-1-1

**Published:** 2006-03-13

**Authors:** Neil R Smalheiser

**Affiliations:** 1 Department of Psychiatry, University of Illinois, Chicago, USA

## Abstract

The *Journal of Biomedical Discovery and Collaboration *was created to provide, for the first time, a unified forum to consider all factors that affect scientific practice and scientific discovery – with an emphasis on the changing face of contemporary biomedical science. In this endeavor we are bringing together three different groups of scholars: a) laboratory investigators, who make the discoveries that are the currency of the scientific enterprise; b) computer science and informatics investigators, who devise tools for data analysis, mining, visualization and integration; and c) social scientists, including sociologists, historians, and philosophers, who study scientific practice, collaboration, and information needs. We will publish original research articles, case studies, focus pieces, reviews, and software articles. All articles in the *Journal of Biomedical Discovery and Collaboration *will be peer reviewed, published immediately upon acceptance, freely available online via open access, and archived in PubMed Central and other international full-text repositories.

## Introduction

"The completion of the human genome project has ushered in a new era in which biology has become an information science."

Thus did Insel et al. [1] succinctly articulate one of the key transitions of biomedical science as we move into the 21^st ^century. As well, research and training are becoming less organized along traditional disciplines (such as pharmacology) and more organized according to problem areas (such as cancer research). Efforts are increasingly devoted, not to testing specific hypotheses, but to *searching for *promising hypotheses; for example, there are many ideas on what causes schizophrenia, but none are formulated so precisely that any particular experiment can currently test or falsify them. An increasingly important source of new discovery involves data mining – analyzing and re-analyzing raw data that reside in genomic and other databases. Just as the solo medical practitioner has largely been replaced by medical organizations, so is the solo investigator becoming incorporated into large multidisciplinary teams (Table 1).

**Table 1:** 20th century vs. 21st century biomedical science

20th Century	21st Century
discipline centered	problem centered
hypothesis testing	hypothesis searching
collect only the data needed to test an hypothesis	collect extra data needed for the future
data archiving	data mining (discovery in databases)
individual scientists and their fiefdoms	flexible, collaborative, multidisciplinary teams

The *Journal of Biomedical Discovery and Collaboration***(JBDC) **was created to provide, for the first time, a unified forum to consider all factors that affect scientific practice and scientific discovery – with an emphasis on the changing face of contemporary biomedical science. In this endeavor we are bringing together three different groups of scholars:

▪ Laboratory investigators, who make the discoveries that are the currency of the scientific enterprise.

▪ Computer science and informatics investigators, who devise tools for data analysis, mining, visualization and integration.

▪ Social scientists, including sociologists, historians, and philosophers, who study scientific practice, collaboration, and information needs.

In the world of sports, different disciplines work together closely so that elite athletes can break Olympic records: There are those who devise the tools (e.g., footwear), those who run the races (the athletes) and those who study athletic performance (exercise physiologists and nutritionists). Yet, the three groups studying scientific practice have operated largely in isolation from each other – having appointments in different colleges, attending different meetings and publishing in different journals. The *Journal of Biomedical Discovery and Collaboration *will provide a venue for discussion and community-building that does not exist elsewhere.

## Article types

### Research articles

These include scholarly studies of scientific practice, information needs, tool development, scientific rhetoric, bibliometrics, data representation methods, and other related topics. Researchers are also encouraged to submit **'discovery notes'**, which describe literature-based discovery or data mining findings that follow systematic methodology and that make clear, testable, nontrivial experimental predictions that deserve the attention of a wide community of scientists.

### Case studies

These describe the structure and programs of individual laboratories, research groups or training programs – how they attempt to foster discovery and collaboration, how this is monitored, lessons learned, and roadblocks to productivity.

### Focus articles

These focus attention on important issues and aim to provoke discussion either by the phrasing of new hypotheses, alternative interpretations of existing work, or by the presentation of new proposals for tackling existing problems. They also provide an opportunity for scientists to publish **'discovery diaries'**, which document the "unexpurgated" story behind a specific experimental study that was previously published in the peer-reviewed literature. What really happened, in what order? What were the relative roles of factors such as hypothesis, policy, collaboration, chance, error, rhetoric, critical pieces of information, and new methods?

### Reviews

These are comprehensive, authoritative, descriptions of any subject within the scope of the journal. These articles are usually written by opinion leaders that have been invited by the Editorial Board.

### Software articles

These describe tools designed to enhance productivity, data mining, creative thinking, data synthesis, and collaborative work. The tools must be available to the public, and need not necessarily be specific to scientific work but should be applicable to scientists. The article must contain some formal evaluation, either of system performance or of user behavior.

## Peer review policy

Peer review is more important than ever. Although online journals are not forced to reject papers due to space limitations, authors and readers alike deserve to have confidence that papers published in the JBDC will be readable and will meet high standards of quality. The JBDC is supported by an outstanding international Editorial Board [2]. All prospective papers, regardless of article type, will be submitted online to the main editorial office and subject to peer review. Reviewers, who are asked to provide anonymous reviews within three weeks of receipt, will consider whether the paper is novel, scientifically sound, topically relevant, balanced, coherent, complete and shows adequate literary quality and scholarship. Papers will receive at least two reviews. In case of a split decision, additional reviews may be sought or an editorial decision may be made.

## Advantages of publishing in an open access journal

Why publish in the *Journal of Biomedical Discovery and Collaboration*? The first reason is that articles will have high visibility. We intend the JBDC to become the preferred venue for the best papers discussing scientific practice and the factors that affect scientific discovery. Second, papers will be published immediately upon acceptance – blazing fast compared to some established print journals where the backlog may be 12–14 months. Third, and perhaps most important, open access articles have a much higher circulation than any traditional disciplinary or specialty journal. Open access ensures that anyone in the world can read the full-text of articles without cost, regardless of their library holdings or subscriptions, both now and in the future. Papers appearing in the *Journal of Biomedical Discovery and Collaboration *will truly have multidisciplinary and international scope. They will be archived permanently in PubMed Central and other international repositories [3]. If Mendel's work had appeared in an open access journal instead of in a regional publication with limited circulation, he would have been much more widely read and the field of genetics might have been launched decades earlier.

It takes courage to submit an article to a new journal based on a new model of open access publishing – probably the same kind of courage it takes to undertake bold experiments in a new field of investigation. We invite you to embark on this adventure with us!
